# Time lag model for batch bioreactor simulation accounting the effect of micro-organism mortality

**DOI:** 10.1080/13102818.2014.993111

**Published:** 2015-01-07

**Authors:** Andrey Zahariev, Hristo Kiskinov, Angel Angelov, Stoyan Zlatev

**Affiliations:** ^a^ Faculty of Mathematics and Informatics, University of Plovdiv, Plovdiv, Bulgaria; ^b^ Department of Biotechnologies, University of Food Technologies, Plovdiv, Bulgaria

**Keywords:** Monod model, micro-organism cultivation, dynamics of micro-organism populations, batch bioreactor, delay equations

## Abstract

In the present work, a generalization of the classical model of Monod accounting the influence of both delayed and instant mortalities on the dynamics of the micro-organism population is proposed. The model was analysed and compared with respect to its quality and applicability for simulation of the cultivation process of micro-organisms. Existence of a unique global positive solution of the Cauchy problem for the proposed model is proved and explicit relations between the decay parameters and the nutrition substrate concentration are obtained. These mathematical results allow us to calculate the nutrient substrate concentration which guarantees that the biomass concentration is maximal for every specific type of taxonomic groups of micro-organisms (bacteria, yeasts).

## Introduction

It is well known that the Monod-type microbial growth models describe adequately bioprocesses appearing in bioreactors and this explains why the Monod-type models are still actual from the theoretical as well as practical point of view.[1] The classical model of Monod [2] of aerobic periodic cultivation of micro-organisms (bacteria, yeasts)(1) 
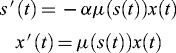
where 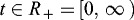, and 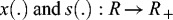 are the concentrations of micro-organisms and the substrate, respectively, has been studied in details by many authors.[1–6] The function 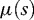 is the intrinsic specific rate of micro-organism population growth and the parameter 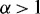 is called economic coefficient (rate of yield). Note that 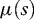 is designed to reflect the limiting influence of the substrate on the microbial growth. It is practically established that the model of microbial growth (1) with monotonously increasing functions of Monod type 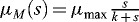 or of Webb type 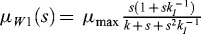, where 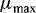 (maximal specifically possible producing rate), 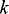 (constant of half saturation) and 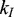 (inhibition constant) are positive constants, adequately describes the dynamics of this process at certain favourable conditions permitting the micro-organisms actively to produce specific enzymes, which are necessary for assimilation and dissimilation of the nutrient substrates. Thus the micro-organisms reproduce themselves at the maximal possible rate 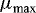. However, that small or large amount of substrate may have an inhibiting (decreasing) effect on the specific rate of microbial growth. In order to reflect this phenomenon in model (1), Haldane [7] and Andrews [3] have suggested the unimodal functions 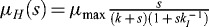 and 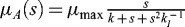, respectively. These functions are also similar and special cases of the Webb [8] function 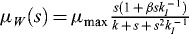 which is unimodal too, when 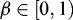. Besides, 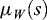 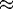 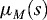 if 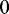 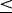 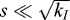. It means that the parameter 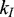 determines in a way the inhibitory phase of the population growth.[6] The basic properties and the graphs of all the four functions are given in [9] where the system(2) 
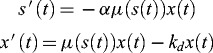
introduced in [10] is under consideration. Here, 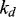 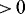 is the specific rate of decay of the micro-organism population. The necessity of models of the kind (2) arises due to unfavourable conditions in the bioreactor. Theoretical and computational analysis of model (2) is fulfilled in [10]. This includes establishing of explicit dependencies between 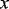 and 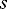 for all four above-mentioned functions as well as between 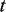 and 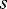 for the first three of them when 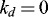. In a previous work,[11] we study the delay analogue of (2), namely(3) 
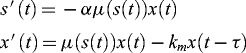
with an initial condition(4) 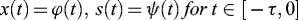
where 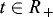, 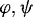: 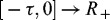, 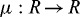 and 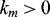 is the specific rate of decay of the micro-organism population. The model (3) was proposed under the reasonable assumption that the individuals of every kind of population have their specific average lifetime 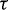 in the bioreactor, which implies that the population decay at the moment 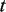 is directly proportional to the micro-organism quantity at the moment 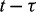.

## Materials and methods

### Materials

Our mathematical model is applicable for all types of bioreactors for aerobic periodic cultivation of micro-organisms (bacteria, yeasts).

### Methods

Since the time of V. Volterra, functional-differential equations (FDEs) are widely used to model biological processes. The transmission of control signals in biological systems is related to such long processes as birth, growth (development) and death. Because of this, the evolution of biological systems depends in an essential way on the whole previous history, and can be modelled in general only by FDEs. Moreover, using FDEs allows us to take into account various insecure factors such as finite lifetime and interaction time; inhomogeneity of the populations lifetime; finite acceptance time for external signals and finite time for elaborating counteractions; pollution effects, resulting in additional mortality with time delay; and spatial environmental heterogeneity. The importance of the aftereffects in population dynamics, and the new effects stipulated by it, determines the practical reason to create delay models which are used to control processes of microbiological growth of cells and production of a useful product. We consider one of them, describing the periodical aerobic reproduction of micro-organisms.

## Results and discussion

### Statement of the problem

From the biotechnological point of view, we presume that the micro-organism mortality is one of the most significant factors influencing successful micro-organism cultivation. Therefore, it is very important to create models which take into account more precisely the micro-organism mortality impact on the population dynamics. In the present paper, we follow this direction and study a combination of models (2) and (3) of the kind(5) 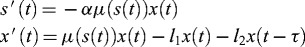

(6) 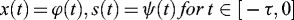
where 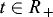, 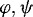: 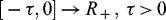, 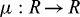, 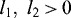 are the specific rates of decay of the micro-organism population. The model (5) describes more precisely the impact of the microorganism mortality for different kinds of micro-organism populations in comparison with the models (2) and (3) taking into account not only the micro-organism mortality in the same moment, but also that in a previous moment. It means that the individuals of every kind of population have their own specific average lifetime 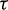 in the bioreactor. It is clear from the biological point of view that the micro-organism mortality in the moment 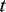 is caused in general by natural reasons, i.e. it is proportional to the quantity of those micro-organisms that have begun their lives in the moment 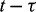 described by the term 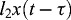 included in the model (5). Thus, we take into account the influence of both (instant and delayed) micro-organism mortalities on the population dynamics. Under biological reasons, one may suppose 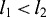 (a majority of the micro-organism population will die after the expiry of its average lifetime 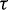), but in our exposition below we will not do that.

In our consideration, we make the following assumption: the material composition is uniform in the reactor and intracellular, while nonuniform space distribution is ignored.

Our basic purpose in this work is to give an explicit answer of the following two practical questions which play an important role in the aerobic periodic cultivation of micro-organisms:

1. For every choice of the function 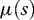, how to calculate practically the minimal concentration of the nutrient substrate 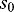 which is necessary to start an increasing micro-organisms reproduction for some period.
2. How to establish practically that the biomass concentration is maximal, i.e. the cultivation process enters in the stationary phase.

Let 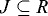 be an arbitrary interval. For every function 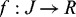, we set by definition 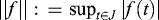 if 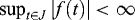. We shall denote by 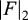 the Euclidean norm of 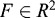, by 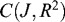 the set of all continuous vector functions 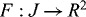 and by 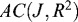 the set of all vector functions 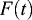 which are absolutely continuous on every closed subinterval of 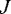. For the vector function 
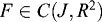
 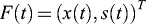, we set by definition 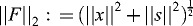 if 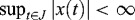 and 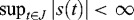. Further, we shall use the following definitions:

Definition 2.1. Vector function 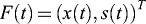, 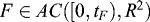, 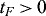 is said to be a solution of the initial value problem (IVP) (5)–(6) in the interval 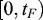 if it satisfies Equation (5) for almost all 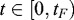 and the initial condition (6) for 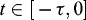.

Definition 2.2. A solution 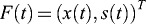 of the IVP (5)–(6) in the interval 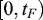 is said to be positive (nonnegative) in the interval 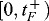, 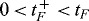 if 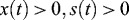 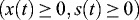 for all 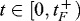.

Definition 2.3. We will say that the property *P* is ultimately fulfilled for some function 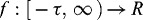 if there exists a point 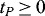, such that for the function 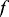 the property *P* holds for each 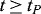.

The basic tasks concerning the model (5) to be solved in the present paper are the existence and uniqueness of a solution of the IVP (5)–(6), analysis of the dynamics of 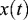 when the nutrient substrate diminishes on a finite or infinite period and studying the influence of the correlation between the parameter 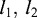 and the values of the function 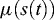 on the dynamics of 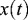.

### Main results

We denote by (H) the following conditions:

(H1). 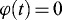 for 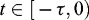 and 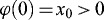.
(H2). 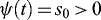 for 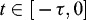.
(H3). There exists 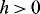 such that the function 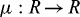 is nondecreasing, continuous and bounded for 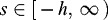 and 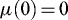.

Lemma 1. Let the conditions (H) be fulfilled.

Then there exists a function 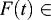 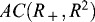 which is the unique solution of the IVP (5)–(6) in 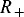.

**Proof.** Denote by 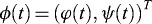 the initial vector function and define for each 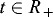 the function(7) 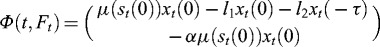

where 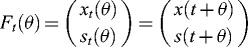, 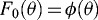 for 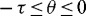.

Let us denote by 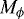 the set of all vector functions 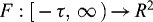, such that 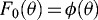, 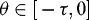 and the restriction 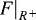 is a continuous vector function. We will denote by 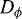 the set 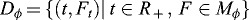 equipped with the metric function 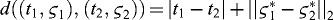, 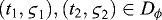 (see [12] Chapter 3, Subs. 2.4), where 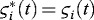 for all 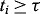 and if 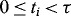, then 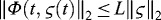. We set 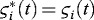 for 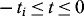 and 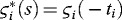 for 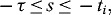 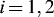.

From the conditions (H), it follows that if the function 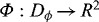 is defined by (7), then the map 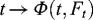 is continuous for any 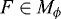. Moreover, for every fixed 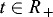, the function 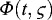 is continuous in every function 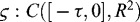. Let 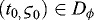 be an arbitrary point, 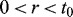 and consider the neighbourhood 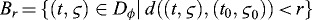. Then, there exists a constant 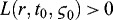 such that the inequality 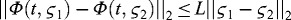 holds for every two points 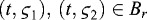. Since 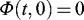, 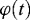 is uniformly continuous in 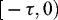, 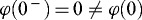 and 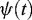 is uniformly continuous in 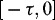, then there exists a point 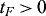 such that the IVP (5)–(6) has a unique solution on the interval 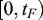. The function 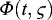 defined by equality (7) is sublinear: that is, for each point 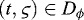, the inequality holds. Therefore, 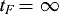 (see [12] Chapter 3, Subs. 2.2–2.4).

Theorem 2. Let the conditions (H) be fulfilled.

Then for every solution 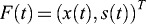 of the IVP (5)–(6) for which there exists a point 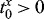 such that 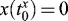 and 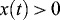 for 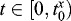, then 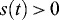 for each 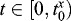.

**Proof.** Assume there exists a point 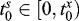 such that 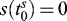 and 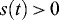 for 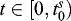. Then, it follows from (5) that 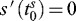 and consequently 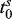 is either an inflection point or a point of local minimum for 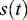. Since 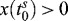, then there exists 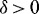 such that 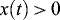 for 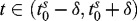. From the first equation of (5) it follows that 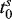 cannot be neither an inflection point nor a point of local minimum for 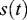.

Corollary 3. Let the conditions (H) be fulfilled.

Then for every solution 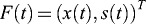 of the IVP (5)–(6) for which there exists a point 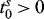 such that 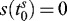 and 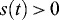 for 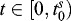 there exists a point 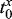 such that 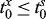, 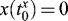 and 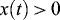 for 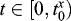.

**Proof.** Assume there does not exist a point 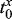 such that 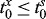 and 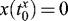. Then, we obtain that 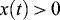 for 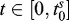, which contradicts the conclusion of Theorem 2.

Theorem 4. Let the conditions (H) be fulfilled.

Then for every solution of the IVP (5)–(6) there exists a point 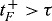 such that the solution 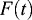 is positive and 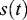 is decreasing for 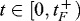.

**Proof.** Let the function 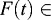 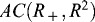 be a solution, existing according to Lemma 1, in the interval 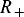. From conditions (H) it follows that there exists a point 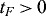 such that 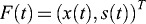 is a positive solution in the interval 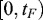. It is easy to see that from (5) it follows 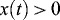 for 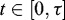. Now assume there exists a point 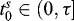 such that 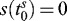 and 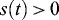 for 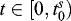. From (5) it follows that 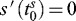 and from condition (H3) it follows that 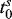 is either an inflection point for 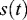 or 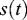 has minimum in the point 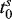 which is impossible because from (5) it follows that 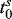 can be neither an inflection point, nor a point of local minimum for 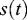. Therefore, 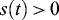 for 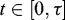. Then, there exists a point 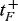 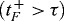 such that 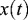 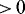 for 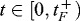 and Theorem 2 implies 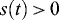 for 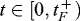. From conditions (H) and (5), it follows that 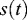 is decreasing for 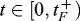.

Let us denote by 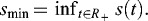

Theorem 5. Let the conditions (H) be fulfilled.

Then, for every solution 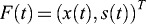 of the IVP (5)–(6) we have 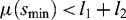.

**Proof.** Assume that 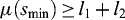. Then, from condition (H3) it follows that 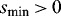. Since 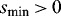 and 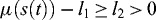 for 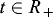, then from Theorem 4 it follows that the inequalities 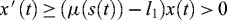 are obviously true for 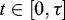 and therefore, 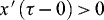 and 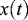 is strictly increasing in the same interval. From (1.5) and Theorem 4 it follows that the inequalities
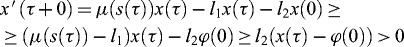

hold. Assume there exists a point 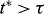 such that 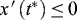 and denote 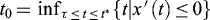. Then we have 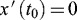 and 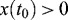. Condition (H3), Theorem 2 and the assumption 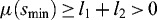 imply the inequalities 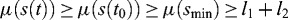 and therefore the following inequality(8) 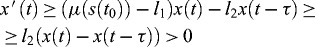
holds for 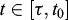. Considering that 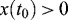 and 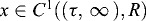, from inequalities (8) it follows 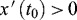 which contradicts 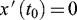. Therefore, 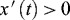 for each 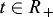 and the first equation of (5) implies that 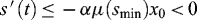. Consequently, the function 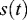 is negative and decreasing for all sufficiently large 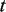 which contradicts the assumption that 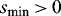.

Theorem 6. Let the following conditions be fulfilled:

1. Conditions (H) are fulfilled.

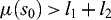
.

Then for any positive solution 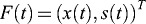 of the IVP (5)–(6) in 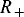, the following equalities 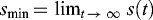 and 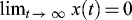 are valid.

**Proof.** Let 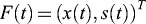 be a positive solution of the IVP (5)–(6). Then, Theorem 5 implies the inequalities 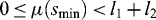. From (5) it follows that 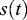 is a positive decreasing function for each 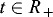 and consequently 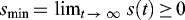. Since in virtue of Theorem 5 we have that 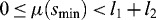, then there exists a unique point 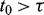 such that 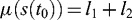 (apparently 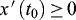 ). Therefore, either 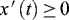 for each 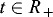 which is impossible, or there exists a point 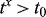 such that 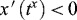. If we denote by 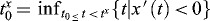, then we have 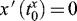. Suppose that there exists a point 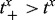 such that 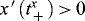 and denote 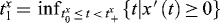. Then, evidently, 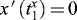 and 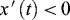 for 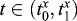.

Consider the case 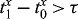. Then, for each 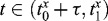 for which 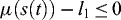 it follows from (5) that 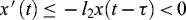 and since 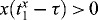, therefore we have 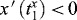 which is a contradiction. In the case when 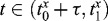 and 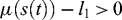, the following inequalities(9) 
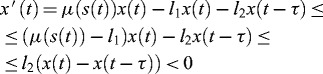



hold. From (9) it follows that 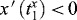 which is impossible.

Consequently, 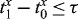 and let 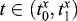. Then, 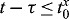 and from (5) for which 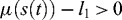, we obtain
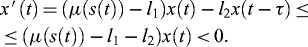



In the case when 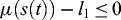, the inequalities 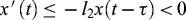 hold and therefore 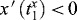 which is impossible. Then, we ultimately have that 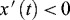 and therefore 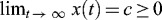. Let us assume that 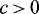. Since 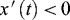, ultimately then it follows from (5) that 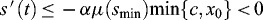 and consequently, we have 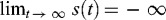 which is a contradiction. Therefore, 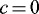.

## Discussion

The proposed model (5) is analysed and compared from mathematical point of view with respect to its quality and applicability for simulation of the cultivation process of micro-organisms. This analysis includes proof of existence of a unique global positive solution of the Cauchy problem (5)–(6) (Lemma 1). The biological sense of Theorem 2 and Corollary 3 is that if the concentration of the nutrient substrate in some finite moment becomes equal to zero, then it is impossible to have live micro-organisms after this moment. Moreover, Theorem 6 implies that if the nutrition substrate vanishes (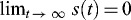 ), then the concentration of the live micro-organisms vanishes too 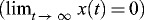. Thus, the model (5) describes the real process more adequately in comparison to the classical model (2) where the nutrition substrate can vanish (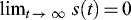 ), but the concentration of the live micro-organisms stay positive (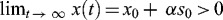 ) and even increases, which is impossible from the biological point of view.

The biological sense of Theorem 5 and the second equation from the model (5) is that the inequality(10) 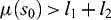
implies increasing reproductive rate of the micro-organism population (at least for period 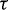 ) and there exists a moment 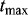 such that the biomass concentration is maximal and(11) 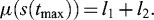



The importance of these relations for the practice is that they give explicit (computational) answers to the questions (1) and (2).

The inequality (10) allows for every specific choice of the function 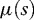 practically to calculate the minimal concentration of the nutrient substrate 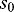 which is necessary for an increasing bacterial concentration at least for period 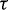.

From the equality (11), we can calculate the critical concentration of the nutrition substrate 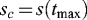 which guarantees that the biomass concentration is maximal. It is enough to measure periodically the nutrient substrate concentration until it reaches the critical level 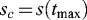. Solve Equation (11) with respect to *s*: 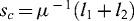


Let us explain this when, for example ,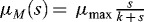. It follows from (10) that if 
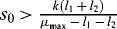
, then we will have increasing bacterial concentration at least for period 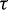 and when the nutrient substrate concentration 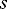 reaches, according to (11), the critical level 
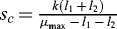
, then the biomass concentration is maximal.

## Conclusion

In this work, a model of aerobic periodic cultivation of micro-organisms (bacteria, yeasts) is introduced and studied. It is more precise comparing to previous models.

Our model allows us to calculate practically the minimal initial concentration of the nutrient substrate, which is necessary to start an increasing micro-organism reproduction for a given period, for every specific rate of micro-organism population growth. Moreover, it allows with a simple measuring of the concentration of the nutrient substrate to establish when the biomass concentration is maximal, i.e. the cultivation process enters in the stationary phase.
